# Index of microcirculatory resistance predicts long term cardiac systolic function in patients with STEMI undergoing primary PCI

**DOI:** 10.1186/s12872-021-01887-w

**Published:** 2021-02-02

**Authors:** Yu Qi, Rong Gu, Jiamin Xu, Lina Kang, Yihai Liu, Lian Wang, Jianzhou Chen, Jingmei Zhang, Kun Wang

**Affiliations:** 1 Department of Cardiology, Nanjing Drum Tower Hospital, The Affiliated Hospital of Nanjing University Medical School, Nanjing, 210008 China; 2 Department of Cardiology, Nanjing Drum Tower Hospital, Clinical College of Nanjing Medical University, Nanjing, 210008 China

**Keywords:** The index of microcirculatory resistance, Acute ST-segment elevation myocardial infarction, Primary percutaneous coronary intervention, Cardiac systolic function

## Abstract

**Background:**

To evaluate the predictive value of the index of microcirculatory resistance (IMR) for long-term cardiac systolic function after primary percutaneous coronary intervention (pPCI) in patients with acute anterior wall ST-segment elevation myocardial infarction (STEMI).

**Methods:**

A total of 53 acute anterior wall STEMI patients were included and followed up within 1-year. IMR was measured to evaluate the immediate intraoperative reperfusion. IMR > 40 U was defined as the high IMR group and ≤ 40 U was defined as the low IMR group. Left ventricular ejection fraction (LVEF) was measured by echocardiography at 24 h, 1 month, 3 months, and 1 year after PCI to analyze the correlation between IMR and cardiac systolic function. Heart failure was estimated according to classification within one year.

**Results:**

The ratio of TMPG (TIMI myocardial perfusion grade) 3 (85.7% vs. 52%, *p* = 0.015) and STR (ST-segment resolution) > 70% (82.1% vs. 48%, *p* = 0.019) were significantly higher in the low IMR group. The LVEF in the low IMR group was significantly higher than that in the high IMR group at 3 months (43.06 ± 2.63% vs. 40.20 ± 2.67%, *p* < 0.001) and 1 year (44.16 ± 2.40% vs. 40.13 ± 3.48%, *p* < 0.001). IMR was negatively correlated with LVEF at 3 months (r = − 0.1014, *p* = 0.0040) and 1 year (r = − 0.1754, *p* < 0.0001).

**Conclusions:**

The IMR showed significant negative correlation with the LVEF value after primary PCI. The high IMR is a strong predictor of heart failure within 1 year after anterior myocardial infarction.

## Background

Acute ST-segment elevation myocardial infarction (STEMI) is one of the most common causes of death in both the developed and developing world [1]. Immediate coronary revascularization by pPCI within 90 min is the gold standard reperfusion therapy for patients with acute STEMI [2]. Despite successful reperfusion of the epicardial coronary artery, a significant proportion of patients with STEMI treated with pPCI fail to achieve adequate myocardial reperfusion due to microvascular system injury and dysfunction [3]. Persistent microvascular dysfunction has been associated with worse long-term cardiac function [4].The current metric for quantifying microvascular injury post-STEMI is cardiac magnetic resonance imaging (CMR), which is not readily available during reperfusion in the catheterization laboratory [5]. In this regard, the index of microcirculatory resistance (IMR), an invasive physiological index and readily available method with extensive clinical evidence, might be used in the evaluation of microvascular function during pPCI [6]. An IMR > 40 U measured after STEMI has been shown to predict cardiac complications, HF readmission, and mortality [7–9]. The aim of this retrospective study with prospectively enrolled patients with acute anterior wall STEMI was to evaluate the predictive value of IMR for long-term cardiac systolic function after pPCI.

## Methods

### Study design and population

The current study represents a retrospective analysis of patients with acute arterial wall STEMI. Patients were prospectively enrolled in Nanjing Drum Tower Hospital from June 2015 to July 2016 (Chinese Clinical Trial Registry, ChiCTR-ICR-15006964). Details about the RCT trial have been reported previously [10]. The target study population was eligible for participation if they were between 18 and 75 years of age, had anterior wall STEMI and chest pain within 12 h, received emergence PCI and presented TIMI grade 0–1 grade during invasive coronary angiography (CAG). The exclusion criteria were as follows: (1) patient undergoing cardiopulmonary resuscitation; (2) patient with heart function grade 3 or 4 (Killip classification) and systolic blood pressure < 90 mmHg; (3) with a history of old myocardial infarction or history of coronary artery bypass grafting (CABG); (4) LAD unsuited stent implantation; (5) patients had thrombolysis before pPCI; (6) had contraindication of adenosine triphosphate (ATP); (7) had a history of liver or renal function dysfunction; (8) had major bleeding events during the past 6 weeks; (9) unable to provide informed consent; and (10) had pregnancy or life span < 1 year. Heart failure was defined as hospitalization because of signs and symptoms of heart failure with noninvasive imaging findings and a discharge diagnosis of heart failure.

### Procedure

#### PCI

Before PCI, all the patients took aspirin 300 mg and P2Y12 (clopidogrel 600 mg or ticagrelor 180 mg). Coronary angiography was performed using standard techniques and the final decision for PCI was at the discretion of the operators. In brief, a guide catheter (5F to 7F) was used to engage the coronary artery, and a pressure–temperature sensor guidewire was used to measure physiologic indices. After wire crossing, 6F Export AP (Medtronic, USA) thrombus aspiration was performed on high thrombus burden patients. Immediately after successful implantation of drug-eluting stents (DES), TIMI and TMPG were recorded at 30 frames per second. The index of microcirculatory resistance (IMR) was detected by fractional flow reserve (FFR) detector (St. Jude Medical, St. Paul, MN, USA).

#### IMR

Maximal hyperemia was induced by adenosine (140 µg/kg/min) intravenously administered into the median cubital vein. A 0.014-in floppy Certus Pressure Wire (St. Jude Medical, St. Paul, USA) and modified Pressure Wire-4 software (Radi Medical Systems, Sweden) were used to measure distal coronary pressure and temperature. We placed a pressure wire into the distal LAD and administered ATP to the Pa 15% decline. Pd, Pa, and FFR were measured and 3 consecutive thermodilution curves were obtained by brisk injection of 3 ml of room temperature saline into the coronary artery, thereby enabling the calculation of mean transit time (Tmin). IMR was calculated by Pd × Tmin [11]. IMR > 40 U was defined as the high IMR group, and ≤ 40 U was defined as the low IMR group [7, 9]. Cardiac function estimated by ejection fraction (EF) was evaluated by 2-dimensional and 3-dimensional ultrasound during 24-h, 1 month, 3 months and 1 year of follow-up. Heart failure was estimated by NYHA classification and followed with in 1 year.

#### Therapy and clinical events

Standard therapy for coronary heart disease was administered before and after the procedure accordingly (aspirin, P2Y12, angiotensin converting enzyme inhibitor/angiotensin receptor blocker (ACEI/ARB), β-blocker, statin). Clinical events were obtained at outpatient clinic visits or by telephone contact when needed. The primary outcome was the all-cause mortality or any revascularization events. The secondary endpoint was 1-year MACE, defined as a composite of worsened heart failure, recurrent angina pectoris, recurrent acute myocardial infarction, or cardiac death.

### Statistical analysis

In addition to the study design, the key factor that will influence the power of the retrospective analysis is the sample size. In this research, we have estimated that if the median EF value at 1 year in IMR > 40 U group is 40.13 (SD, 3.48) and that in ≤ **40** U group is 44.16 (SD, 2.40), and loss of follow-up is 0.1, then 9 subjects per group would be needed. All discrete or categorical variables are presented as numbers and relative frequencies (proportions), which were compared with the chi-square test or Fisher’s exact test, as appropriate. Continuous variables are presented as the mean ± SD or median (interquartile range) according to their distributions, which were compared with Student’s t test. Nonnormally distributed continuous variables were compared with the Kruskal–Wallis test. Correlations between variables were expressed with Pearson r. Cox proportional hazard regression models were used to determine predictors of the clinical end points. Statistical analysis was performed with Prism 7, and *p* < 0.05 was considered statistically significant.

## Results

### Baseline clinical characteristics

**Fig. 1:**
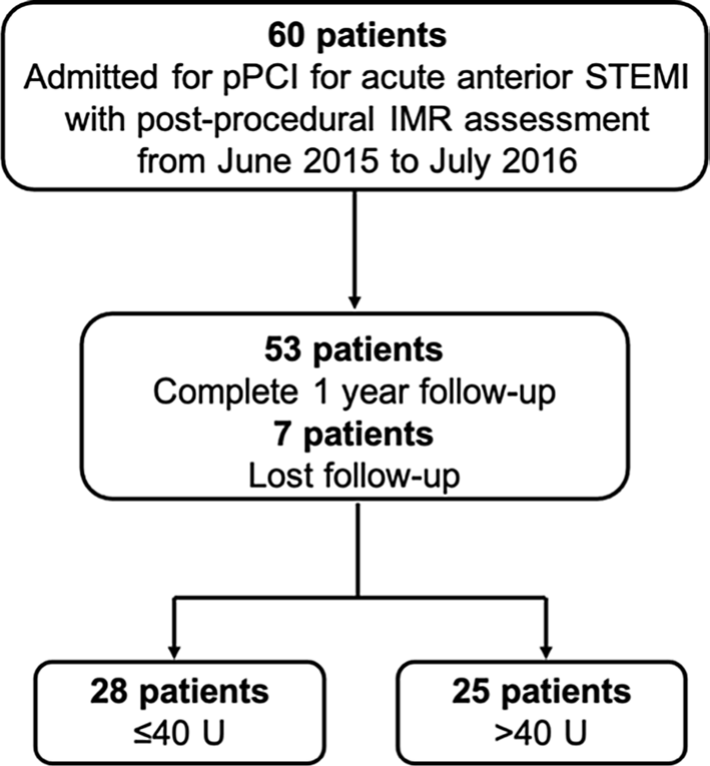
Study flow chart. pPCI, primary percutaneous coronary intervention; STEMI, ST-segment elevation myocardial infarction

A total of 53 anterior wall STEMI patients who were followed up without any death for 1 year were included in the present analysis (Fig. 1). The baseline clinical characteristics are presented in Table 1. In this study, the high IMR was defined as > 40 U, and the low IMR group was ≤ 40 U. There was no difference between the two groups in age, hypertension, diabetes, smoking, hyperlipemia, ischemic time or other cardiovascular risk factors.

**Table 1:** Baseline clinical characteristics

	IMR ≤ 40 U (n = 28)	IMR > 40 U (n = 25)	*p* value
Male	23 (82.1)	23 (92.0)	0.295
Age (years)	56.2 ± 10.5	60.2 ± 10.2	0.168
Hypertension	17 (60.7)	17 (68.0)	0.775
Diabetes mellitus	7 (25.0)	8 (32.0)	0.761
Smoking	21 (75.0)	13 (52.0)	0.095
Hyperlipemia	4 (14.3)	6 (24.0)	0.488
Ischemic time (min)	246 ± 180	293 ± 150	0.293
D–B (min)	81.0 ± 24.2	74.6 ± 24.1	0.340
*Admission BP* SBP (mmHg) DBP (mmHg)	130.4 ± 15.9 80.4 ± 16.5	126.6 ± 15.0 80.4 ± 11.0	0.377 0.986
*Periprocedural medication*			
Aspirin P2Y12 ACEI Statin β-blocker	28 (100.0) 28 (100.0) 28 (100.0) 28 (100.0) 28 (100.0)	25 (100.0)	–
		25 (100.0)	–
		25 (100.0)	–
		25 (100.0)	–
		25 (100.0)	–
Hemoglobin (g/L)	124.6 ± 7.7	125.6 ± 7.4	0.609
Blood glucose (mmol/L)	6.5 ± 1.7	6.5 ± 2.2	0.637
T-CHO (mmol/L)	5.4 ± 1.7	5.0 ± 1.6	0.321
LDL-C (mmol/L)	2.7 ± 0.8	2.7 ± 0.8	0.586
HbA1c (%)	5.7 ± 0.8	6.1 ± 1.4	0.214
BNP (pg/ml)	218.6 ± 136.5	254.5 ± 220.1	0.542
IMR	22.4 ± 6.6	43.6 ± 3.7	< 0.001

Values are mean ± SD or n (%)

D-B, door-balloon; SBP, systolic blood pressure; DBP, diastolic blood pressure; ACEI, angiotensin converting enzyme inhibitor; T-CHO, total cholesterol; LDL-C, low density lipoprotein; HbA1c, glycosylated hemoglobin; BNP, B-type natriuretic peptide; IMR, index of microvascular resistance

### Angiographic characteristics

There was no difference in angiography parameters including LAD occlusion, drug eluting stent (DES) and balloon dilation between the two groups, except for a significantly higher percentage of TMPG 3 (85.7% versus 52%, *p* = 0.015) and a higher STR > 70% ratio (82.1% versus 48%, *p* = 0.019) among patients with low IMR (Table 2).

**Table 2:** Angiographic characteristics

	IMR ≤ 40 U (n = 28)	IMR > 40 U (n = 25)	*p* value
LAD occlusion			
Proximal Middle	13 (46.4) 15 (53.6)	11 (44.0) 14 (56.0)	1.000 1.000
DES			
Number	1.2 ± 0.4	1.2 ± 0.4	0.827
Length (mm)	29.5 ± 12.3	31.0 ± 15.0	0.691
Balloon dilation			
Predilation Postdilation	7 (25.0) 14 (50.0)	12 (48.0) 12 (48.0)	0.095 1.000
TIMI 3 grade	26 (92.9)	20 (80.0)	0.234
TMPG 3 grade	24 (85.7)	13 (52.0)	0.015
STR > 70%	23 (82.1)	12 (48.0)	0.019

Values are mean ± SD or n (%)

LAD, left anterior descending; DES, drug eluting stent, TIMI, thrombolysis in myocardial infarction, TMPG, STR: ST-segment resolution

### Cardiac function analysis

**Fig. 2:**
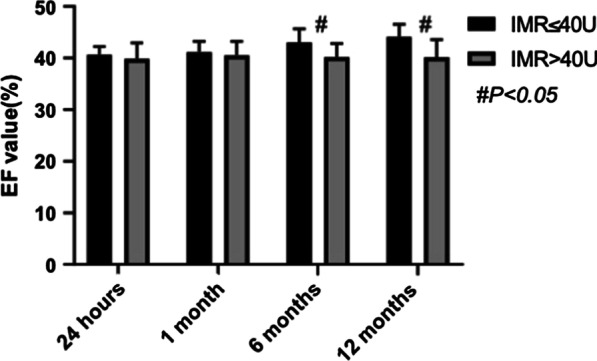
Follow-up EF values between the IMR ≤ 40 U group and the IMR > 40 U group. Values are mean ± SD

Cardiac function estimated by EF was not different between the two groups at 24 h (40.66 ± 1.58% versus 39.87 ± 3.10%, *p* = 0.242) or 1 month (41.16 ± 2.12% versus 40.52 ± 2.76%, *p* = 0.342). However, the EF value in the low IMR group was significantly higher than that in the high IMR group at 3 months (43.06 ± 2.63% versus 40.20 ± 2.67%, *p* < 0.001) and 1 year (44.16 ± 2.40% versus 40.13 ± 3.48%, *p* < 0.001) after PCI (Table 3 and Fig. 2).

**Table 3:** Cardiac function analysis

	IMR ≤ 40 U (n = 28)	IMR > 40 U (n = 25)	*p* value
EF value at 24 h	40.66 ± 1.58	39.87 ± 3.10	0.242
EF value at 1 month	41.16 ± 2.12	40.52 ± 2.76	0.342
EF value at 3 months	43.06 ± 2.63	40.20 ± 2.67	< 0.001
EF value at 1 year	44.16 ± 2.40	40.13 ± 3.48	< 0.001

Values are mean ± SD or n (%)

EF, ejection fraction

### Correlation analysis between IMR and LVEF

**Fig. 3:**
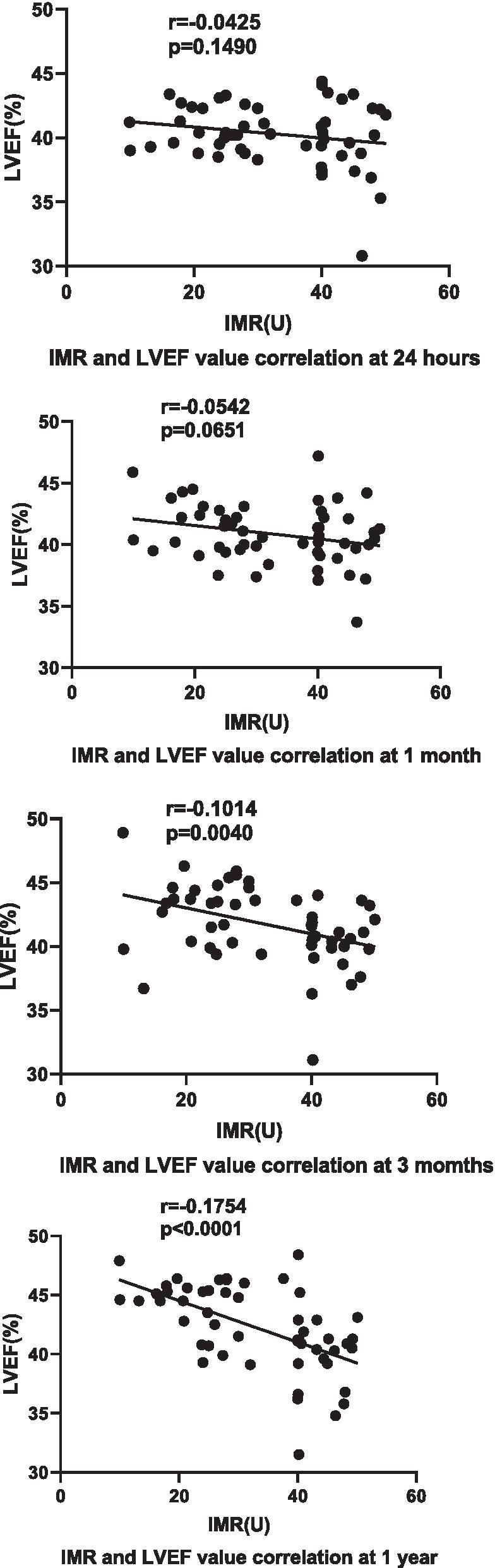
Correlations analysis between IMR and LVEF. LVEF, left ventricular ejection fraction

IMR had no significant correlation with left ventricular ejection fraction (LVEF) at 24 h (r = − 0.0425, *p* = 0.1490) and 1 month (r = − 0.0542, *p* = 0.0651) but had a negative correlation with LVEF at 3 months (r = − 0.1014, *p* = 0.0040) and 1 year (r = − 0.1754, *p* < 0.0001) (Fig. 3).

### The incidence of clinical events

**Fig. 4:**
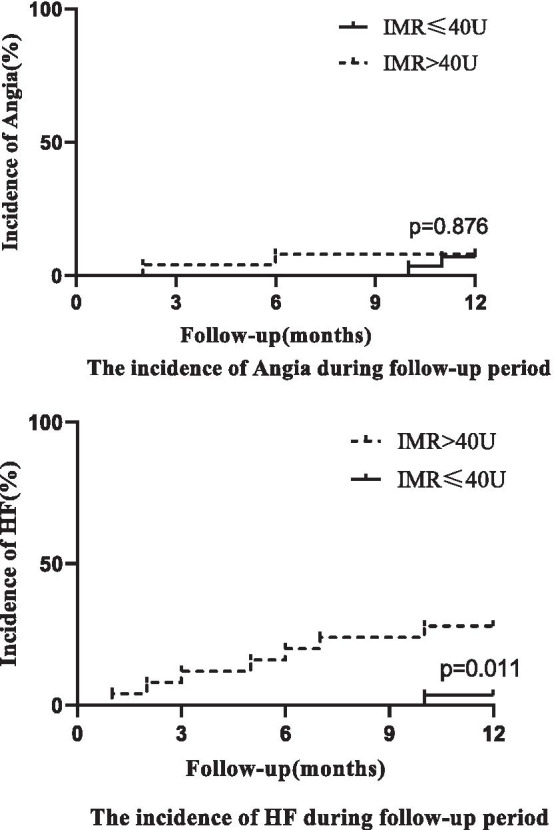
The incidence of heart failure and angina during the follow-up period

There wasn’t any death among patients. Angina pectoris and heart failure were only major adverse cardiac events. None of the patients had a recurrent myocardial infarction. The incidence of angina was not significantly different between the two groups (*p* = 0.876). However, there were 7 patients diagnosed with heart failure, which was classified II according to NYHA classification. A higher cumulative incidence of heart failure occurred in the IMR > 40 U group than in the ≤ 40 U group at the 1-year follow-up (*p* = 0.011). This result suggested that a higher IMR was associated with heart failure (Fig. 4).

## Discussion

The main findings of our study are: (1) There is a significant negative correlation between IMR and LVEF in patients with anterior wall STEMI at three months and 1-year after primary PCI; (2) The IMR is an independent predictor of heart failure at 1-year after primary PCI.

The purpose of pPCI in acute STEMI was to improve myocardial perfusion and cardiac function [10]. However, half of all treated patients opening a culprit epicardial vessel have a suboptimal result from pPCI, with impaired persistent microvascular dysfunction, such as reperfusion injury or microvascular obstruction (MVO), which predicts worse outcomes in the longer term [12]. Immediate assessment of suboptimal myocardial reperfusion could enable the prompt identification of high-risk patients who could potentially benefit from additional therapy [6].

IMR is an invasive, readily available, quantitative and reproducible, wire-based measure of the minimal achievable coronary microvascular function that exploits the ability of one of the commercially available pressure wire systems to measure both coronary pressure and flow [11, 13]. In patients with STEMI, a distinct advantage of IMR is that it can be measured immediately after pPCI in the catheterization laboratory simultaneous with fractional flow reserve (FFR) and thereby allow the independent interrogation of both the epicardial artery and the coronary microvasculature at an early enough time when therapeutic intervention may alter the clinical course of patients [14].

There are some other predictors of post -PCI EF in ant MI patients. Hayıroğlu and colleagues assessed the prognostic value of precordial total Q wave amplitude to precordial total R wave amplitude ratio (Q/R) on 354 patients with first acute anterior MI treated with primary PCI, which indicated that Q/R is an independent prognostic factor for in-hospital mortality, cardiogenic shock and MACE [15]. Syntax score II (SSII) connects clinical variables with coronary anatomy. Hayıroğlu et al. also reported SSII in 492 patients with STEMI complicated with cardiogenic shock treated with pPCI provide an independent prognostic marker of in-hospital outcomes [16]. While single center nature of these two studies was an important limitation due to the selection bias.

Some studies found that the CK peak concentration with an IMR value below 40 U was relatively low, the ventricular wall motion score could be significantly improved at 3 months, and better clinical prognosis was shown in the short-term follow-up [17, 18]. Previous studies have shown that TMPG 3 could independently predict the level of myocardial perfusion [19]. In this study, the ratio of TMPG 3 and STR greater than 70% was significantly higher in the low IMR group, which further confirmed the association between low IMR and relatively sufficient myocardial perfusion.

We further analyzed the correlation between IMR and LVEF values, and the study showed that IMR and EF values were negatively correlated at 3 months and 1 year after PCI, with statistically significant differences, which was consistent with research of McGeoch [20] showing that IMR was a strong predictor of EF and infarct size. Fearon et al. measured IMR in STEMI patients and showed that IMR was a strong predictor of recovery of left ventricular systolic function after 3 months [17]. Studies have also shown that IMR measured after PCI in patients with acute anterior wall myocardial infarction can predict the myocardial survival, microcirculatory function recovery and cardiac function after 6 months [18, 21, 22], which is consistent with the results of this study.

Finally, IMR is useful for the early identification of long-term microvascular dysfunction in patients with acute STEMI. Current evidence supports the feasibility of studies using IMR as a predictor of microvascular dysfunction and as a measure of treatment effect. The results of this study confirm that IMR can predict the long-term cardiac systolic function of acute anterior wall myocardial infarction, and the optimization of an emergency PCI strategy based on IMR can improve microcirculatory function, thus improving the long-term cardiac systolic function. These data illustrate an insight from measuring IMR as a tool to guide the stratification of patients for adjunctive therapeutic strategies in acute ST-segment elevation myocardial infarction.

## Conclusions

The results of our study show a significant negative correlation of IMR and LVEF after primary PCI during long term follow-up. Patients with IMR > 40 U are at increased risk of the development of heart failure with in first one year after anterior wall STEMI, which indicated that high IMR may be a useful independent predictor for post-myocardial infarction cardiac function.

## Data Availability

The datasets generated and/or analyzed during the current study are available from the corresponding author on reasonable request.

## Abbreviations

ACEI: Angiotensin converting enzyme inhibitor
ARB: Angiotensin receptor blocker
ATP: Adenosine triphosphate
BNP: B-type natriuretic peptide
CABG: Coronary artery bypass grafting
CAG: Coronary angiography
CMR: Cardiac magnetic resonance imaging
D-B: Door-balloon
DBP: Diastolic blood pressure
DES: Drug-eluting stents
FFR: Fractional flow reserve
HbA1c: Glycosylated hemoglobin
HF: Heart failure
IMR: The index of microcirculatory resistance
LAD: Left anterior descending
LDL-C: Low density lipoprotein cholesterol
LVEF: Left ventricular ejection fraction
MACE: Major adverse cardiac events
pPCI: Primary percutaneous coronary intervention
SBP: Systolic blood pressure
STEMI: ST-segment elevation myocardial infarction
STR: ST-segment resolution
T-CHO: Total cholesterol
TIMI: Thrombolysis in myocardial infarction
TMPG: TIMI myocardial perfusion grade
